# Net Costs Due to Seasonal Influenza Vaccination — United States, 2005–2009

**DOI:** 10.1371/journal.pone.0132922

**Published:** 2015-07-31

**Authors:** Cristina Carias, Carrie Reed, Inkyu K. Kim, Ivo M. Foppa, Matthew Biggerstaff, Martin I. Meltzer, Lyn Finelli, David L. Swerdlow

**Affiliations:** 1 Office of Science and Integrated Programs, Centers for Disease Control and Prevention, Atlanta, Georgia, United States of America; 2 IHRC.INC, Atlanta, Georgia, United States of America; 3 Influenza Division, Centers for Disease Control and Prevention, Atlanta, Georgia, United States of America; 4 Battelle Memorial Institute, Atlanta, Georgia, United States of America; 5 Division Of Preparedness And Emerging Infections, Centers for Disease Control and Prevention, Atlanta, Georgia, United States of America; University of Western Australia, AUSTRALIA

## Abstract

**Background:**

Seasonal influenza causes considerable morbidity and mortality across all age groups, and influenza vaccination was recommended in 2010 for all persons aged 6 months and above. We estimated the averted costs due to influenza vaccination, taking into account the seasonal economic burden of the disease.

**Methods:**

We used recently published values for averted outcomes due to influenza vaccination for influenza seasons 2005-06, 2006-07, 2007-08, and 2008-09, and age cohorts 6 months-4 years, 5-19 years, 20-64 years, and 65 years and above. Costs were calculated according to a payer and societal perspective (in 2009 US$), and took into account medical costs and productivity losses.

**Results:**

When taking into account direct medical costs (payer perspective), influenza vaccination was cost saving only for the older age group (65≥) in seasons 2005-06 and 2007-08. Using the same perspective, influenza vaccination resulted in total costs of $US 1.7 billion (95%CI: $US 0.3–4.0 billion) in 2006-07 and $US 1.8 billion (95%CI: $US 0.1–4.1 billion) in 2008-09. When taking into account a societal perspective (and including the averted lost earnings due to premature death) averted deaths in the older age group influenced the results, resulting in cost savings for all ages combined in season 07-08.

**Discussion:**

Influenza vaccination was cost saving in the older age group (65≥) when taking into account productivity losses and, in some seasons, when taking into account medical costs only. Averted costs vary significantly per season; however, in seasons where the averted burden of deaths is high in the older age group, averted productivity losses due to premature death tilt overall seasonal results towards savings. Indirect vaccination effects and the possibility of diminished case severity due to influenza vaccination were not considered, thus the averted burden due to influenza vaccine may be even greater than reported.

## Introduction

Influenza vaccination has been recommended for all persons aged six months of age and older since 2010 [1]. In the U.S. each year, seasonal influenza has been estimated to cause 31.4 million outpatient visits, >200000 hospitalizations, 3000–49000 deaths, and is responsible for 44.0 million days lost ([2] estimations based on 2003 population) [1,3]. While most morbidity occurs in persons ≥65 years of age, all age groups are affected. Morbidity and mortality vary per season, depending on the types, subtypes and phenotypes of the circulating influenza viruses, levels of prior natural immunity, as well as on the antigenic match between the seasonal vaccine with the circulating viruses.

Economic evaluations of influenza vaccination in the United States have generally not addressed the particularities of specific seasons, instead relying on decision trees in which the probability of medical outcomes is not season specific, and wide confidence intervals relay seasonal variation in coverage and vaccine effectiveness [4–6]. Therefore, such estimates cannot be used to for season specific economic evaluation of influenza vaccination by age group. While cohort studies have been conducted relating actual outcomes from longitudinal studies, they are only specific to certain age-groups and not general to the entire population [7,8].

The goal of this study is to provide season-specific economic evaluations of the net cost of the influenza vaccine, using age-specific estimates of vaccine coverage, vaccine effectiveness, and health outcomes averted by influenza vaccine. A model that uses monthly vaccine coverage data and seasonal vaccine effectiveness estimates has recently become available to estimate the magnitude of seasonal averted outcomes for the seasons 2005–06 to 2008–09 (the season 2009–2010 was marked by an influenza pandemic, and was excluded from the analysis) [9–11]. We combine this model with updated cost information to calculate net costs from influenza vaccination in the United States. The next sections detail the methods, cost model, cost estimates, and the results obtained.

## Methods

We measured the net costs of influenza vaccination, from both payer and societal perspectives. The total costs due to influenza vaccination include the overall costs of administering the vaccine, and the costs associated with the vaccination’s adverse effects. However, such costs do not take into account the benefits of the vaccination program, and thus overestimate the program’s costs. We measured the net costs of influenza vaccination by also taking into account the burden averted by vaccination. The payer perspective takes into account averted direct medical costs, whereas, from a societal perspective, both averted direct medical costs and averted productivity losses due to influenza vaccination are considered. Influenza vaccine recipient time costs are also considered from a societal perspective. To note, a cost analysis does not address whether the intervention’s cost is worth the social benefit. The following sections detail the cost model and the estimation of cost parameters.

### 2.1 Cost Model

The total costs of influenza vaccination, in season *t*, for a given age group, consist of the sum of administration costs (*AdmCost*), plus the sum of costs due to influenza vaccination adverse effects:

$$TC_{t,Age}=AdmCost_{t,Age}+\sum_{j}AdvEffect_{j,t,Age}.CostAdvEffect_{j,Age}$$ (1)

where the age cohorts are 6m-4 years, 5–19 years, 20–64 years, and 65 and older ages; and the summation over adverse effects include injection site reaction, systemic reactions such as fever, myalgias, fatigue, malaise, and headaches leading to a doctor visit, anaphylaxis, and Guillain-Barre syndrome.

Net costs (NC) due to influenza vaccination correspond to the difference, in season *t*, between vaccination associated costs and the averted costs due to vaccination:

$$NC_{t,Age}=AdmCost_{t,Age}+\sum_{j}AdvEffect_{j,t,Age}.CostAdvEffect_{j,Age}-\sum_{i}AvertedHealthOutcomes_{i,t,Age}.CostHealthOutcome_{i,Age}$$ (2)

Where the summation over averted health outcomes includes deaths, hospitalizations, medically attended cases, and ill, not medically attended cases. The number of averted hospitalizations, medically attended, and not medically attended cases per season and age-group has been published and is taken from Kostova et al, [11] (Table 1, S1 Appendix). To calculate averted outcomes (hospitalizations, medically attended cases, and ill, not medically attended cases) the observed risk of a given outcome is first extrapolated to an entirely unvaccinated population. The number of observed outcomes is then extracted from this number, resulting in the estimated number of averted cases. This method assumes that vaccination of one individual averts influenza outcomes for that individual alone. To calculate risks, the number of outcomes is determined using estimated multipliers relating the number of hospitalizations to the number of medically attended patients, and the number of not medically attended cases. Averted deaths were taken from a study taking into account age-specific influenza-associated excess mortality ([2,12], Table 1, S2 Appendix).

**Table 1:** Averted outcomes per season, and ranges.

Season/ Age groups		Deaths	Hospitalizations	Medically Attended Cases	Ill, not Medically Attended Cases
05–06	AgeGroup				
	0–4	4(0–9)	1,960(1084–3,429)	118,073(66,081–214,000)	161,094(88,375–274,358)
	5–19	2(0–4)	522(281–918)	79,952(43,564–146,080)	109,889(58,666–187,864)
	20–64	143(11–292)	1,890(1054–3,215)	117,667(66,351–207,808)	160,603(88,862–265,384)
	65 ≥	2094(141–6872)	8,484(4,698–14,830)	230,437(128,709–417,093)	309,738(170,410–527,159)
06–07	AgeGroup				
	0–4	16(5–30)	2,216(1330–3,640)	133,527(80,762–226,718)	182,179(108,632–291,819)
	5–19	8(3–14)	539(297–931)	82,539(45,810–146,801)	113,444(62,084–191,753)
	20–64	174(63–293)	1,752(1102–2,805)	109,049(69,214–181,233)	148,839(92,969–231,728)
	65 ≥	2406(712–5782)	4,393(2,718–7,089)	119,322(74,175–200,011)	160,386(98,897–251,339)
07–08	AgeGroup				
	0–4	16(2–32)	3,333(2,073–5,364)	200,777(124,972–336,387)	273,932(170,373–427,675)
	5–19	10(4–20)	1,043(642–1,706)	159,769(98,392–269,500)	219,591(134,994–350,864)
	20–64	409(194–799)	6,020(3,992–9,311)	374,732(249,901–603,264)	511,467(337,784–767,252)
	65 ≥	4647(1715–13689)	20,225(13,414–31,107)	549,329(367,817–883,761)	738,372(486,246–1,096,809)
08–09	AgeGroup				
	0–4	34(19–55)	3,737(2,333–5,982)	225,112(141,780–376,297)	307,132(190,586–475,791)
	5–19	20(13–29)	1,584(989–2,568)	242,677(151,815–409,603)	333,542(208,027–524,260)
	20–64	339(209–509)	3,029(1,962–4,803)	188,540(121,493–311,448)	257,337(167,336–395,580)
	65 ≥	3575(1574–7338)	4,081(2,515–6,617)	110,844(68,586–186,931)	148,988(91,537–234,350)

Averted outcomes were assumed to be distributed according to a truncated normal distribution, with standard deviation = mean/10, where the mean corresponds to the point estimates. Values adapted from [11, 12]. For the sensitivity analyses, a lognormal and triangular distribution were also explored.

We further distinguished between averted cases among high vs. non-high risk people (S3 Appendix). High risk conditions include asthma, neurological and neurodevelopmental conditions, chronic lung disease, heart disease, blood disorders, endocrine disorders, kidney disorders, liver disorders, metabolic disorders, a weakened immune system due to disease or medication, and morbid obesity [13]. These conditions increase the likelihood that the patient will require costlier treatments [14]. For each medically attended health outcome (medically attended case, hospitalization, hospitalization ending in death) the share of averted cases involving high risk and non-high risk medical conditions was assumed to be equal to the analogous share of medical outcomes occurring each year, which was informed by the literature [2]; i.e., we assumed that the probability of becoming vaccinated was equal for high and non-high risk patients. If this is not the case, and groups at risk of developing more complicated medical conditions are also more likely to be vaccinated and consequently averting or attenuating costlier medical conditions (as it seems to be the case [15]), costlier medical conditions will be underrepresented in averted medical outcomes. We therefore performed one way sensitivity analysis to understand how variation in the share of averted cases that was high risk impacted the results. Empirical confidence intervals for final net costs were obtained by numerical approximation methods (Monte Carlo simulation using 500000 draws from the probability distributions, implemented with @Risk, v5.7.1, Palisade Corporation NY, a spreadsheet based software).

### 2.2 Cost of health outcomes

Costs were calculated for different health outcomes attributed to influenza for cohorts characterized by different age and underlying risk condition (Table 2). The health outcomes resulting from influenza infection considered were: death following hospitalization; hospitalization resulting in discharge; medically attended case not involving hospitalization; illness not medically attended. The population was divided into broad age categories that are distinct with respect to economic activity and health care-related costs: 6 months-4 year olds; 5–19 year olds; 20–64 year olds; and above 65 years. Given that high-risk conditions are strongly associated with costlier procedures, and longer duration of influenza episodes, for each age cohort, costs were calculated for individuals with non-high-risk, and with high-risk conditions for influenza complications, where the definition of high-risk conforms to ACIP definitions [16,17]. In particular, a given case was identified as non-high-risk if it was characterized by a Pneumonia and Influenza ICD9 code (480, 481, 482, 483, 484, 485, 486, 487.00, 487.10, 487.80, 488, 487), and was not characterized by any high-risk condition ICD-9 code. A high-risk case was identified by a Pneumonia and Influenza ICD9 code and an ICD9 code for a high-risk condition. The share of averted cases that were high risk was assumed to be equal to the relative burden of high risk conditions [2], and so, for each medical outcome, the proportion of averted costs due to high risk cases was the same as the occurring proportion of high risk cases.

**Table 2:** Cost of Influenza Outcomes.

**Hosp. and death**	**Medical costs for NH risk**		**Medical costs for H risk**		**Productivity losses–PVLE** *		
**Age Group**	**Mean**	**Sd**	**Mean**	**Sd**	**Mean**	**Sd**	
0–4	49339	45013	46017	18752	1253537	259839	
5–19	167086	154674	188071	187836	1488118	1050693	
20–64	66174	100023	71826	123238	776866	2900666	
65≥	33011	74178	44806	77873	216738	696982	
**Hospitalization**		**Indirect costs for NH risk**	**Medical costs for NH risk**		**Indirect costs for H risk**	**Medical costs for H risk**	
**Age Group**	VLD**	Productivity losses/ DL	Mean	Sd	Productivity losses/ DL	Mean	Sd
0–4	150	5	9390	17834	7	20928	51476
5–19	150	7	19258	57774	10	40310	89190
20–64	150	8	22556	21714	12	29528	43881
65≥	117	7	12689	24005	10	19099	44164
**Outpatient**		**Indirect costs for NH risk**	**Medical costs for NH risk**		**Indirect costs for H risk**	**Medical costs for H risk**	
**Age Group**	VLD**	Productivity losses/ DL	Mean	Sd	Productivity losses/ DL	Mean	Sd
0–4	150	1	339	697	1	603	1436
5–19	150	1	277	665	2	843	2225
20–64	150	1	422	1376	1	665	2160
65≥	117	2	922	4084	2	2592	6862
**Not Medically attended Case**		**Indirect costs** *	**Medical Costs** *				
**Age Group**	VLD**	Productivity losses/ DL	Mean	Sd			
0–4	150	1	4	3			
5–19	150	0.5	4	3			
20–64	150	0.5	4	3			
65≥	117	1	4	3			

Costs in 2009USD$. Abbreviations: DL—Days lost; VLD—Value of a Lost Day; PVLE—Present Value of Lost Earning; NH/H risk—Non-high risk/ high-risk. Lognormal distribution assumed for Costs. Poisson distributions assumed for Days Lost [2].

*Costs obtained from [2];

**Cost information from [17,21] (See S4 Appendix).

Information on direct medical costs for influenza health outcomes was drawn from a large insurance claims dataset (Truven Health MarketScan Research Databases—MarketScan is a registered trademark of Truven Health Analytics Inc.) for the 2007–2008 influenza season, and updated to $US (year 2009 values) using the medical cost component of the Consumer Price Index [18]. High risk inpatient admissions tend to occur later in the influenza season, and so information was retrieved between the months of August 2007 and May 2008 in order to fully capture the influenza season. The Truven Health MarketScan Research Databases contain individual-level, de-identified, healthcare claims information from employers, health plans, hospitals, Medicare, and Medicaid programs. Insurance claims data have been shown to be a reliable source of cost information [14]. While costs may also be estimated using the accounting systems of different hospitals or care institutions [14,19–21], such measurements are not available for all age-groups, use smaller and geographic-specific samples, and hence show a high variation in final estimates.

#### 2.2.1 Medical Costs

For each risk and age cohort, medical costs were calculated for ill, not medically attended cases; medically attended cases; hospitalized cases; and hospitalizations resulting in death (Table 2). For cases not requiring medical assistance, costs consist of the costs of over the counter medication (assumed to be $US 3). For medically attended cases costs include the costs of outpatient visits, and associated prescription cost per influenza episode. Clustering per influenza episode meant that outpatient visit and drug prescription costs were aggregated over a period in which the interval between consecutive outpatient visits was not more than 15 days. Related drug related prescriptions were considered to be the ones within a fifteen day time window from the outpatient visits.

For hospitalizations, an episode of influenza corresponds to an inpatient admission after which the patient was discharged to home, and drug related prescriptions and outpatient visits occurring at least within 14 days before admission to 30 days after discharge and extending for a period for which there was no interruption of more than 14 days. Total individual costs consist of the summation of the total cost of inpatient admissions, pharmaceutical prescriptions and related outpatient visits. Individual values were then averaged by risk and age cohort. Costs for a hospitalization followed by death were calculated similarly, for a discharge status indicating death.

#### 2.2.2 Indirect Costs

To calculate indirect costs we estimated days lost to illness or recovery, and valued such days using the Present Value of Lost Earnings [22] (Table 2). When possible, number of days was estimated by using the Medstat MarketScan database, and when data was not available, number of days was estimated based on published data [2,23]. For medically attended cases, days lost due to illness were considered to be equal to the number of outpatient visits. For cases involving hospitalizations, days lost due to illness were considered to be the summation of the length of stay of the inpatient admission, and the number of outpatient visits. For not medically attended illness, assumed days lost to illness were based on the literature [2].

Productivity loss was valued using the Present Value of Lost Earnings. To gauge the value lost due to death, tabulated values for lost earnings were used, and updated to 2009 values using the Consumer Price Index for All Items [2,18,22]. For days lost due to illness, for each age cohort, the value of a lost working day was valued using the mean hourly wage, taking into account the share of the population that was unemployed or out of the workforce ([24–26], S4 Appendix). Individuals that are out of the workforce or unemployed can still provide valuable services, and so the lost productivity of the share of the unemployed and out of the workforce population was estimated using the value of an unspecified day as estimated in [22]. As children are likely to require the assistance of a healthy productive individual, the value of days lost to illness in the age groups 6m-4 and 5–19 was considered to be the same as to the value of days lost to illness in the age group 20–64. For the elderly (65≥), the proportion of individuals out of the workforce is much higher than in other age groups, and, again, the value of days lost to illness consisted of a weighted average between the average value of a working day and the average value of an unspecified day [22]. Cost values and associated distributions are depicted in Table 2.

### 2.3 Cost of vaccination

Administration costs include the costs of the vaccine dose, supplies, labor, and overhead (including promotion and advertising). They depend crucially on the setting [5,27]. Costs have been estimated to vary between $US 14.0 per shot in a pharmacy and $US 63.8 per shot for a scheduled appointment in a family size clinic. Other settings include mass vaccination settings such as the workplace, supermarkets, or schools, where administration cost has been estimated at $US 20.6 per shot (all costs updated to 2009 values using the medical cost component of the Consumer Price Index) [5].

Medical settings and mass vaccination settings are used by a majority of individuals aged 18 and above. Over ninety percent of all vaccines are given in a medical setting, in the workplace, or in a store [28]. Working adults and those aged 65≥ are more likely to be vaccinated in a medical setting, but over a third opt to be vaccinated in a mass vaccination setting [28]. For this reason, we considered the mean cost of administering the vaccine to be $US 19.8 ($US 19.6 for those 65≥), equal to a weighted average between the cost of being vaccinated in a medical setting (valued as a walk-in appointment in a corporate clinic at $US 19.1) and the cost of being vaccinated in a mass vaccination setting ($US 20.6) ([5, 27], S4 Appendix). To take into account variability in the choice of the setting, the cost of administering the vaccine was considered to follow a truncated normal distribution varying between $US 14.0 and $US 63.8. Patient time lost was included when measuring net costs from a societal perspective, and varied between $US 2.2 and $US 37.6, with a mean of $US 14.5 for those <65. For those 65≥, recipient time cost varied between $US 2.2 and $US 29.2, with a mean of $US 9. The upper bounds correspond to the value of 2 hours in a doctor’s office, while the lower value corresponds to the lower range considered for time spent in a mass vaccination setting [5]. Vaccine coverage data was taken from the National Health Interview Survey as published previously [11], and assumed to follow a truncated normal distribution (S1 Appendix).

The likelihood and costs of vaccination adverse events was adapted from published data drawn from expert panels and previous retrieval of insurance claims data [4,5], and was assumed to remain constant across seasons (Table 3). Costly vaccination adverse effects include Guillain-Barre syndrome, an autoimmune disorder; anaphylaxis; and systemic reactions such as fever, myalgias, fatigue, malaise, and headaches leading to a doctor visit [29,30].

**Table 3:** Probability and cost of influenza vaccine adverse effects.

Influenza Adverse Events	Age-Group	Probability			Medical costs			Days lost		
		Base-case estimate	Range		Base-case estimate	Range/Sd		Base-case estimate	Range	
**Local reaction**	**-**	0.44	0.03	0.585	-	-		-	-	-
**Systemic reaction**	0–4	0.011	0.0008	0.02	211	502		0.25	-	-
	5–19	0.03	0.0002	0.05	120	326		0.25	-	-
	20–64	0.011	0	0.044	158	554		0.25	-	-
	65 ≥	0.011	0	0.044	306	1952		0.25	-	-
**Anaphylaxis**	0–19	0.00000025	0	0.000001	523	476	578	2.09	1	5
	20 ≥	0.00000025	0	0.0000025	556	411	752	2.37	1	8
**Guillain- Barre Syndrome**	0–19	0.000001	0	0.00001	76190	51221	102782	39.68	7	123
	20 ≥	0.000001	0	0.000002	76190	51221	102782	39.68	7	123

Costs in 2009USD$. Adapted from [4,5,29,30]

## Results

Results varied by season and age group: in season 07–08 for age-group 65≥ influenza vaccination was cost saving, while for season 06–07 and age-group 20–64 years the administration of influenza vaccine resulted in net costs of $US 1 billion (Table 4). Overall, influenza vaccination was cost saving for the older age group, and for a season in which the influenza-associated averted mortality and morbidity was high. While influenza vaccination does not appear to be cost saving for the intermediate age groups, the high averted costs due to influenza vaccination that occurred in season 07–08 in the older age group compensated for the excess costs due to administering the vaccine in the intermediate age groups, resulting in net savings due to influenza vaccination in season 07–08, when taking into account productivity losses. Accounting for both medical and indirect costs (societal perspective), influenza vaccination was not cost saving in 06–07 and 08–09, mild seasons in which the number of averted cases was not high, even though it was cost saving for the older age cohort in all 4 years. It was also not cost saving in season 05–06, in which averted morbidity was comparatively high, but averted mortality was not.

**Table 4:** Net costs of the U.S. influenza vaccination program (in millions), by age group and season: mean values and 95% empirical confidence intervals.

Season	Age Group	Net cost, accounting for Medical Costs Averted	Net cost, accounting for Medical and Productivity Losses Averted
05–06	0–4	109(CS-328)	156(CS-381)
	5–19	227(43–642)	338(147–754)
	20–64	918(407–2604)	1304(412–3019)
	65 ≥	CS(CS-897)	CS(CS-828)
	Total	1205(CS-3582)	1415(CS-4008)
06–07	0–4	136(CS-360)	188(CS-419)
	5–19	285(94–703)	431(228–852)
	20–64	1051(540–2757)	1508(514–3255)
	65 ≥	239(CS-1064)	CS(CS-1022)
	Total	1712(294–4013)	2023(CS-4594)
07–08	0–4	116(CS-357)	155(CS-411)
	5–19	286(CS-716)	438(118–873)
	20–64	911(CS-2679)	1193(CS-3089)
	65 ≥	CS(CS-590)	CS(CS-388)
	Total	417(CS-3270)	CS(CS-3421)
08–09	0–4	98(CS-347)	102(CS-368)
	5–19	274(CS-718)	411(CS-865)
	20–64	1163(493–2912)	1608(CS-3458)
	65 ≥	235(CS-1093)	CS(CS-994)
	Total	1769(146–4149)	1771(CS-4626)

Values in millions 2009USD$. Abbreviation: CS—Cost Saving.

In season 05–06, net savings occurred in the older age group, mainly due to the high number of averted deaths and consequent high indirect costs averted, while in the other age groups the cost of administering the vaccine and the cost of adverse effects was more than the averted cost due to influenza vaccination. In the younger age group the vaccine was not cost saving overall, but net costs ($US 156 million, from a societal perspective) were lower than those in age groups 5–64 years mainly due to the high numbers of averted cases and consequent averted medical and productivity losses.

For season 06–07, when only taking into account medical costs (payer perspective), influenza vaccination was not cost saving for any age group. When taking into account indirect costs (societal perspective), influenza vaccination was cost saving only for the 65≥ age group. Season 06–07 was a relatively mild season, hence the number of averted cases was not high, and averted costs did not exceed the costs of administering the vaccine and the costs of vaccine adverse effects.

In season 07–08, influenza vaccination resulted in overall net savings when considering medical and indirect costs (societal perspective). Again, cost savings occurred in the 65≥ age group, while in the age group 5–19 years, influenza vaccination resulted in net costs of $US 431 million, from a societal perspective. When considering only medical costs (payer perspective), influenza vaccination resulted in net savings only for the older age group, due to the high number of cases averted in this cohort.

Finally, for the season 08–09, and again from a payer perspective (only taking into account medical costs), influenza vaccination did not result in net savings. The magnitude of indirect costs is however high for all age groups, and so, when taking into account productivity losses (societal perspective) influenza vaccination resulted in net savings for the 65≥ age group. Nevertheless, such net savings did not offset administration costs in the intermediate age groups.

### 3.1 Sensitivity Analysis

Our preferred choice for the distribution of averted outcomes was the normal distribution. We quantified model uncertainty by analyzing how results varied with specifying different distributions for averted outcomes. The number of averted outcomes was specified to vary according to triangular, normal, and lognormal distributions. Results (Table 4) were robust to the different specifications. For individual age groups, values were similar when using the lognormal or normal distribution. When using the triangular distribution, mean costs suffered a decrease <10% for most age-groups/seasons. For the evaluation of the season as a total, results were robust to the lognormal distribution. Seasonal net costs generally suffered a decrease >10% when using the triangular distribution, due to the higher probability weight given to the upper bounds of averted outcomes (especially relevant in the older age group).

**Fig 1:**
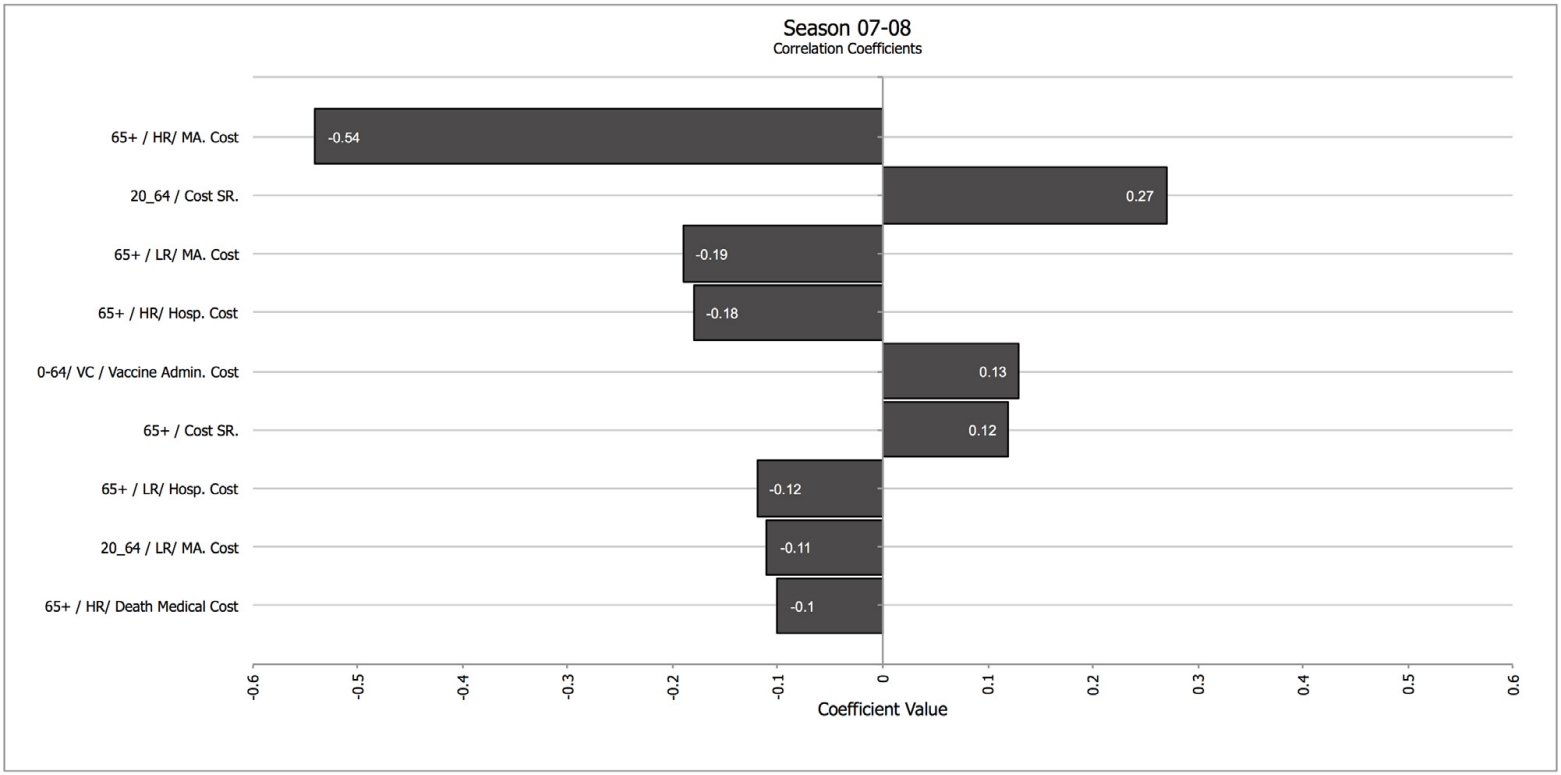
Tornado coefficient for Net Costs (all age cohorts, payer perspective) for season 07–08. Correlation coefficients in the horizontal axis. Abbreviations: Cost SR.–Cost of Systemic Reaction; HR—High Risk; Hosp.–Hospitalization; LR: Low Risk; MA—Medically Attended; VAC—Vaccine Administration Cost. When applicable, the numbers in the left of the factor represent the age cohort the costs refer to. Components with correlation values <0.1 were not included.

**Fig 2:**
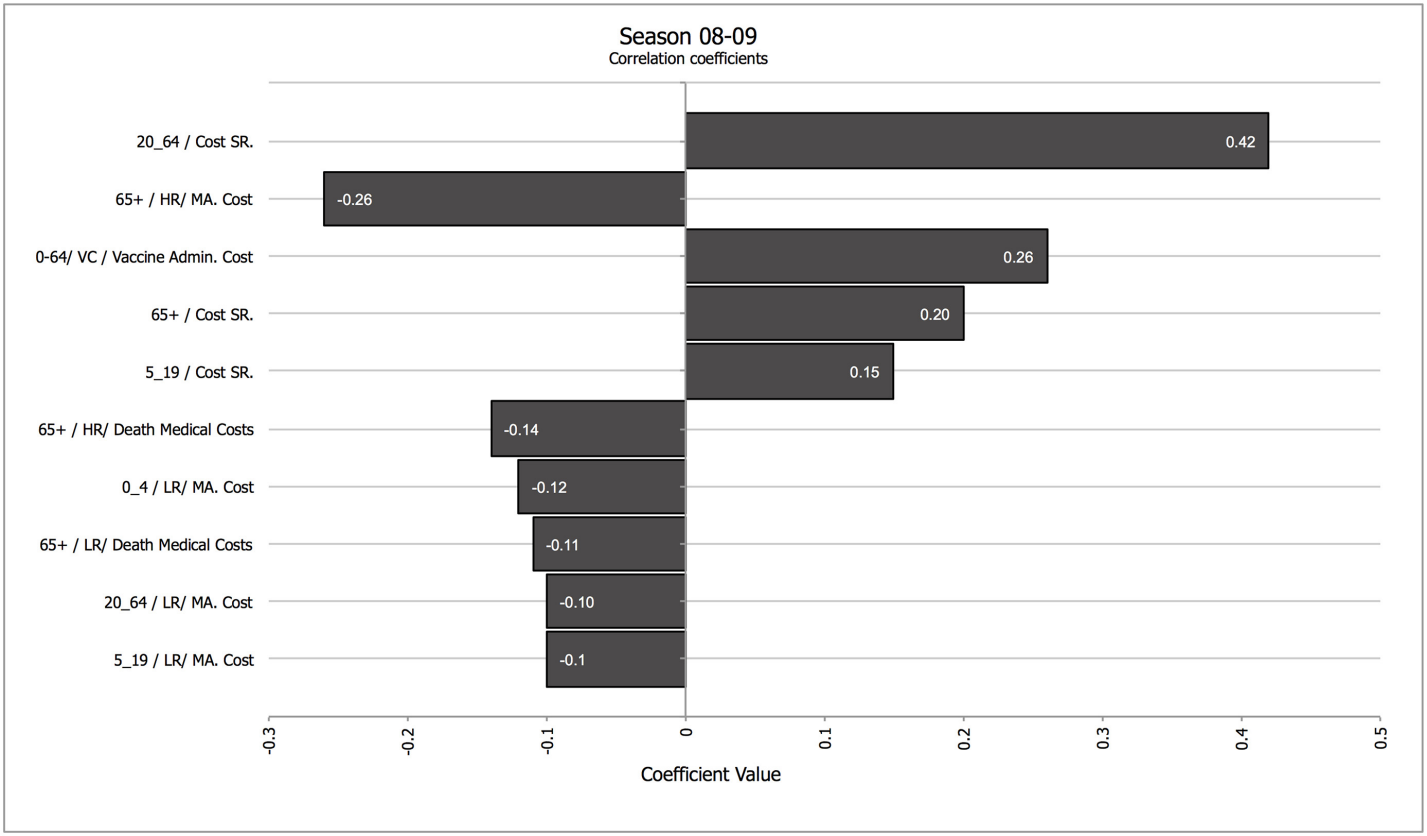
Tornado coefficient for Net Costs (all age cohorts, payer perspective) for season 08–09. Correlation coefficients in the horizontal axis. Abbreviations: Cost SR.–Cost of Systemic Reaction; HR—High Risk; LR—Low Risk; MA—Medically Attended. When applicable, the numbers in the left of the factor represent the age cohort the costs refer to. Components with correlation values <0.1 were not included.

**Fig 3:**
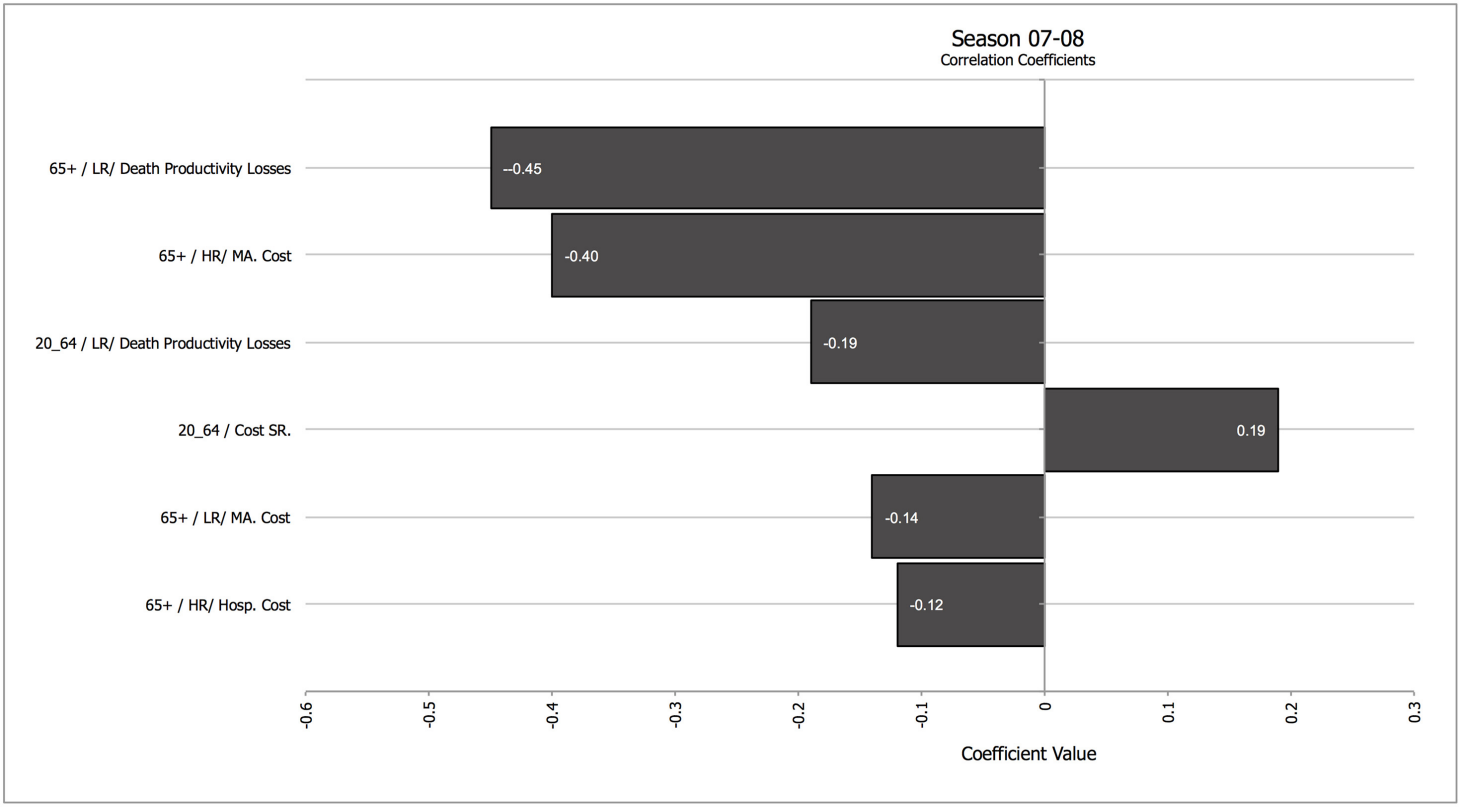
Tornado coefficient for Net Costs (all age cohorts, including productivity losses, societal perspective) for season 07–08. Correlation coefficients in the horizontal axis. Abbreviations: Cost SR.–Cost of Systemic Reaction; HR—High Risk; LR: Low Risk; MA—Medically Attended. When applicable, the numbers in the left of the factor represent the age cohort the costs refer to. Components with correlation values <0.1 were not included.

**Fig 4:**
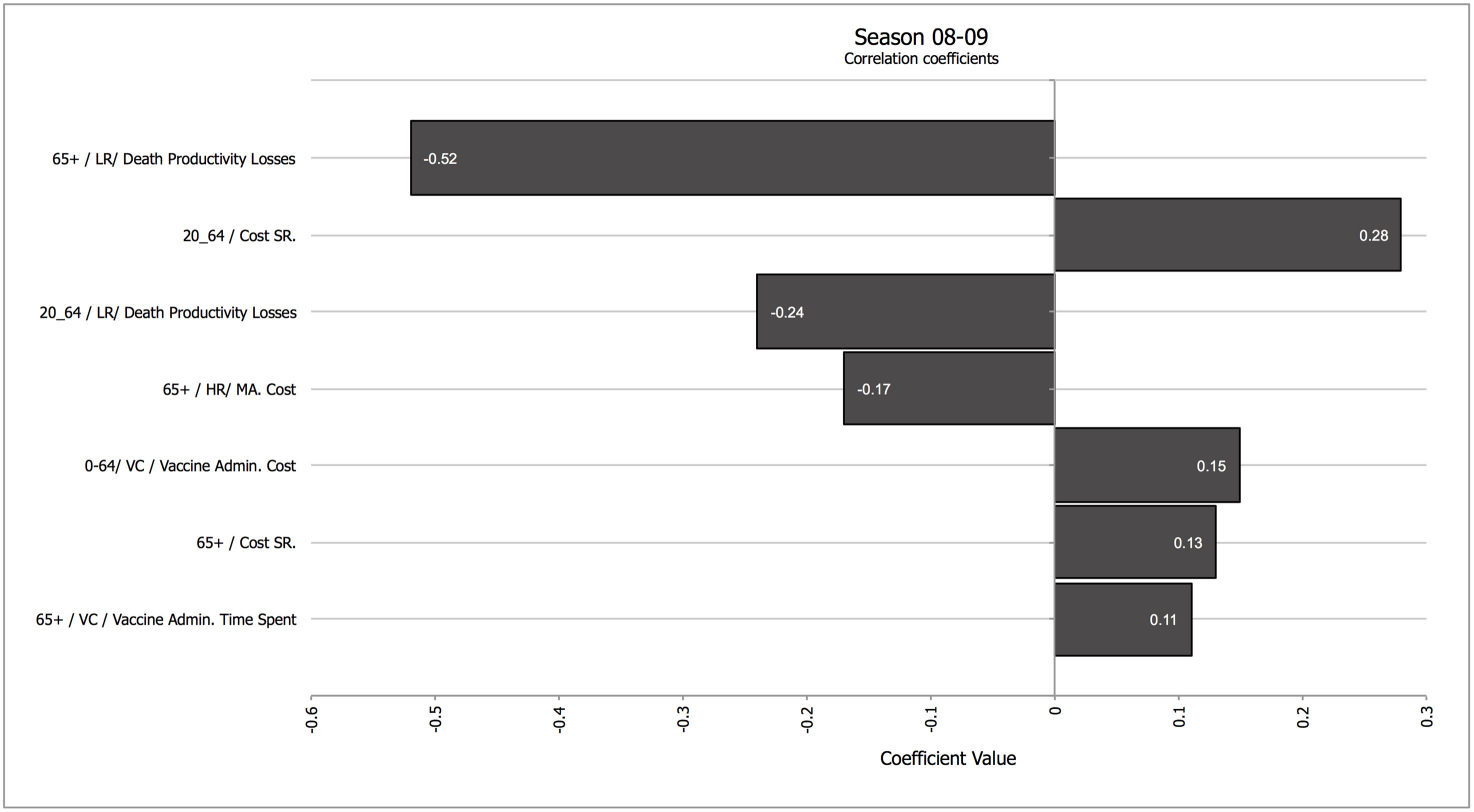
Tornado coefficient for Net Costs (all age cohorts, and including productivity losses, societal perspective) for season 08–09. Correlation coefficients in the horizontal axis. Abbreviations: Cost SR.–Cost of Systemic Reaction; HR—High Risk; LR: Low Risk; MA—Medically Attended; VAC—Vaccine Administration. When applicable, the numbers in the left of the factor represent the age cohort the costs refer to. Components with correlation values <0.1 were not included.

Secondly, we analyzed how different parameters influenced the results in a season in which vaccination had a strong impact (07–08) and in a season in which vaccination had a low impact (08–09). Figs 1 and 2 show a tornado graph depicting the influence of different parameters on net medical costs for season 07–08 and for season 08–09, and the drivers of total averted costs for the same seasons (Fig 3 for season 07–08, and Fig 4 for season 08–09). A tornado graph depicts how overall results vary with the individual components; the size of each bar is proportional to the correlation between variations in each component of the calculation and the final result. Components with higher absolute correlation values are the ones with a higher effect on the final results; components with lower correlation values are not as influential. Negative correlation values for the relationship between a component and the final result mean that as the ingredient increases, the final result decreases. The most significant drivers of net medical cost were the cost of medically attended high risk patients in the elder age group, and the cost of systemic reactions after vaccination in the 20–64 years cohort, for season 07–08; for season 08–09 the drivers were the cost of systemic reactions in the 20–64 years cohort, the cost of medically attended high-risk cases, and the vaccine administration cost.

Figs 3 and 4 show the dominant influence of indirect productivity losses due to death in the older age group in driving net averted costs in all seasons. For season 07–08, the cost of outpatient visits for high-risk elders was also relevant. In both seasons, the cost of adverse effects in the 20–64 years cohort was also important in determining costs averted.

Results were sensitive to variations in the proportion of averted cases that was high risk. Doubling the proportion of averted cases that was high risk (meaning that more cases were averted among the high risk population than those that would be expected if vaccination coverage was the same among high and low risk groups) resulted in a decrease of net costs of approximately 10% in seasons 06–07 and 08–09, from a payer perspective. For seasons in which the averted burden was higher (seasons 05–06 and 07–08), the decrease was higher as well. Averted costs were similar to administration costs for season 07–08, from a payer perspective. For season 05–06 the decrease was up to 20%, from a payer perspective.

## Discussion

We have analyzed the net costs of seasonal influenza vaccination in the United States, from both payer and societal perspectives. Given the high value of averted productivity losses, seasonal influenza vaccination can be cost saving when considered from a societal perspective. However, the averted social costs due to influenza vaccination varied per season, and seasonal net savings occurred only in seasons in which averted mortality and morbidity was high (07–08). In this season, the costs of deaths averted due to the influenza vaccine were very high and tilted the results towards overall net savings. For season 07–08, when productivity losses costs were not included, and only direct medical costs were considered (payer perspective), net savings occurred only in the cohort of individuals aged 65 and above. In milder seasons (06–07 and 08–09), net savings in the social cost of influenza occurred only in the elderly cohort. When considering medical costs only (payer perspective) the vaccine was not cost saving. While the influenza vaccine also caused net savings in the elderly age group, such net savings did not offset other costs. Results were robust to several sensitivity analyses.

Such findings are consistent with other economic analyses of the influenza vaccine that stress the relevance of the social burden of disease when analyzing the results, and the high impact of the influenza vaccine among the elderly [6,8,31]. The vaccine was not found to be cost saving in healthy working adults, in contrast to Prosser et al. (2008) in which the vaccine was found to be cost saving for a general influenza season. This is probably a reflection of the more pessimistic assumptions underlying the present study: Prosser et al (2008) [5] had assumed a vaccination effectiveness of 69% (30%-90%) for adults, while the mean vaccination effectiveness considered in Kostova et al. (2013) [11] for the present seasons was less than 50% on average, with confidence intervals varying between 17% and 79% for selected seasons.

The sensitivity analyses also reflect the influence of vaccination adverse effects in driving net medical and net averted costs, especially in influencing net medical costs in seasons with a low vaccination impact, such as 08–09. In seasons with a higher vaccination impact (such as 07–08), other factors, such as the cost of medically attended high risk patients in the older cohort come into play. However, these results should be interpreted carefully given the extremely limited published evidence on the likelihood of outpatient visit given a systemic reaction such as fever or myalgia.

Results show that influenza vaccination can be cost saving, and highlight the importance of researching the likelihood of adverse effects of vaccination. Results should be interpreted with qualifiers, however. Costing analyses do not take into account measures of effectiveness or social preference. Even if an intervention is costly and not cost saving, it may be worth undergoing if the benefits are deemed to be worth the costs, which we have not analyzed here. Also, this study suffers from three main limitations. First, we did not consider the possibility that the influenza vaccine may mitigate without preventing illness. While this possibility is controversial [32] some studies suggest that influenza vaccination decreased the likelihood of influenza related complications [7,31]. Cases with lower severity could present in a variety of ways: cases for which death was avoided but instead only required a hospitalization or for which the length of the hospitalization was less, or cases that would have required hospitalization but instead only required an outpatient visit. Second, while we only considered that productivity losses due to illness occurred during the days the patient was hospitalized or had an outpatient visit, productivity losses due to illness are potentially higher. Third, we also ignored the possible indirect effects of vaccination i.e. its population effect by which people not vaccinated may be at lower risk for infection. To the extent that this effect may substantially increase the number of averted cases and the consequent magnitude of averted costs, it also suggests that averted costs due to the influenza vaccination may have been underestimated.

We used new methods and season-specific data to assess the seasonal averted cost due to the influenza vaccine. Our results show that by averting potential influenza cases, the influenza vaccine offsets some of the vaccine administration costs, being cost saving in some seasons and for some age-groups. Because this study relies on seasonal data and estimated outcomes instead of season fixed assumptions, it effectively addresses how variation in influenza season characteristics influences economic outcomes in the United States. When averted mortality and morbidity are high, the influenza vaccine results in cost savings, with savings in the elderly cohort offsetting costs incurred in the middle age groups. In milder seasons, the vaccine is cost saving especially in the elderly age group, a group where vaccination coverage is particularly high. While this study uses recent data on the number of averted cases due to the vaccine, it does not take into account cost savings due to lowering the severity of the cases or herd immunity, suggesting that the averted costs due to influenza vaccine are potentially higher.

## Supporting Information

S1 Appendix Inputs and methods to calculate the averted burden. (DOCX)

S2 Appendix Method to calculate averted deaths. (DOCX)

S3 Appendix Share of burden that was high-risk. (DOCX)

S4 Appendix Additional inputs to calculate productivity losses, and the cost of administering the vaccine. (DOCX)
