# Supplementary material for: Net Costs Due to Seasonal Influenza Vaccination — United States, 2005–2009
Source: PLoS One. 2015 Jul 31;10(7):e0132922. doi: 10.1371/journal.pone.0132922 (PMC4521706; doi:10.1371/journal.pone.0132922)
Supplement: S1 Appendix — (DOCX) [file pone.0132922.s001.docx]

**Appendix 1 – Calculation of averted burden**

Table SI-1: Vaccination coverage, vaccine effectiveness, and total population used for the calculation of averted burden, by age group (95% confidence intervals in parentheses).

| Season | Age-Group |  | Vaccine Coverage | | | |  | Vaccine Effectiveness | | | |  | Total Population |
| --- | --- | --- | --- | --- | --- | --- | --- | --- | --- | --- | --- | --- | --- |
| 05-06 | 0_4 |  | 0.349 | (0.305 | - | 0.401) |  | 42.1 | (18.5 | - | 58.9) |  | 18,435,382 |
|  | 5_19 |  | 0.15 | (0.134 | - | 0.169) |  | 42.1 | (18.5 | - | 58.9) |  | 61,588,987 |
|  | 20_64 |  | 0.204 | (0.191 | - | 0.215) |  | 42.1 | (18.5 | - | 58.9) |  | 176,976,709 |
|  | 65≥ |  | 0.66 | (0.625 | - | 0.699) |  | 29.5 | (13.0 | - | 41.2) |  | 36,703,697 |
|  |  |  |  |  |  |  |  |  |  |  |  |  |  |
| 06-07 | 0_4 |  | 0.445 | (0.385 | - | 0.504) |  | 50.5 | (41.7 | - | 57.9) |  | 18,551,515 |
|  | 5_19 |  | 0.201 | (0.172 | - | 0.225 |  | 50.5 | (41.7 | - | 57.9) |  | 61,777,006 |
|  | 20_64 |  | 0.233 | (0.224 | - | 0.248) |  | 50.5 | (41.7 | - | 57.9) |  | 178,997,496 |
|  | 65≥ |  | 0.677 | (0.656 | - | 0.716) |  | 35.4 | (29.2 | - | 40.5) |  | 37,205,916 |
|  |  |  |  |  |  |  |  |  |  |  |  |  |  |
| 07-08 | 0_4 |  | 0.482 | (0.403 | - | 0.549) |  | 47.3 | (39.7 | - | 54.0) |  | 18,829,160 |
|  | 5_19 |  | 0.233 | (0.209 | - | 0.259) |  | 47.3 | (39.7 | - | 54.0) |  | 61,935,681 |
|  | 20_64 |  | 0.262 | (0.25 | - | 0.278) |  | 47.3 | (39.7 | - | 54.0) |  | 180,855,780 |
|  | 65≥ |  | 0.692 | (0.661 | - | 0.728) |  | 33.1 | (27.8 | - | 37.8) |  | 37,867,145 |
|  |  |  |  |  |  |  |  |  |  |  |  |  |  |
| 08-09 | 0_4 |  | 0.51 | (0.404 | - | 0.494) |  | 50.5 | (43.2 | - | 56.9) |  | 19,037,307 |
|  | 5_19 |  | 0.282 | (0.235 | - | 0.277) |  | 50.5 | (43.2 | - | 56.9) |  | 62,045,041 |
|  | 20_64 |  | 0.287 | (0.271 | - | 0.295) |  | 50.5 | (43.2 | - | 56.9) |  | 182,377,351 |
|  | 65≥ |  | 0.696 | (0.654 | - | 0.698) |  | 35.4 | (30.2 | - | 39.8) |  | 38,799,891 |

From [1]. Vaccine coverage and total population were used to calculate administration costs. Vaccine coverage was assumed to follow a truncated normal distribution with standard deviation=mean/10.

**Technical appendix: Methodology description to calculate averted burden**

This appendix describes the methodology used to estimate averted burden in Kostova et al [1].

The methodology consisted of 4 steps:

1. Estimating influenza burden (hospitalizations, medically attended cases, and cases);
2. Estimating rates of illness among susceptible population;
3. Estimating the burden in the absence of influenza vaccination;
4. Calculating the difference between the burden of influenza vaccination and the estimated burden.

**Step 1 –** To calculate influenza burden, a pyramid model was employed. Hospitalizations were first estimated using national surveillance data. The number of cases was then calculated by multiplying the ratio of hospitalization to cases [2]. The number of medically attended cases was calculated as a proportion of the total number of cases.

**Step 2 –** To calculate rates of illness among susceptible population, the susceptible population (“population at risk”) in every month was first calculated. Then, for each outcome (hospitalization, medically attended cases, cases), the outcome’s rate was estimated by dividing the total number of outcomes by the “population at risk”. The monthly “population at risk” was proportional to the share of population that was not effectively vaccinated (that is: population at risk ≈ [population – cases]*[1 – vaccine effectiveness x vaccine coverage] for a given time t).

**Step 3 –** In order to calculate the burden in the absence of influenza vaccination, the rates of influenza illness were multiplied by the population at risk. In the beginning of the season, the population at risk corresponds to the total population. However, as the season progresses, the monthly population at risk is adjusted to increasing vaccine coverage and natural immunity as calculated in step 2.

**Step 4 –** To calculate the averted burden, the difference between the burden of influenza in the absence of vaccination and the estimated influenza burden was calculated.

Monte Carlo simulation was used to calculate empirical confidence intervals.

References:

1. Kostova D, Reed C, Finelli L, Cheng PY, Gargiullo PM, Shay DK, et al. Influenza Illness and Hospitalizations Averted by Influenza Vaccination in the United States, 2005-2011. PLoS One. 2013;8: e66312.
2. Reed C, Anjulo FJ, Swerdlow DL, Lipsitch M, Meltzer MI et al. (2009) Estimates of the prevalence of pandemic (H1N1) 2009, United States, April-July 2009. Emerging Infectious Diseases 15(12):2004-2007.
