# Supplementary material for: Net Costs Due to Seasonal Influenza Vaccination — United States, 2005–2009
Source: PLoS One. 2015 Jul 31;10(7):e0132922. doi: 10.1371/journal.pone.0132922 (PMC4521706; doi:10.1371/journal.pone.0132922)
Supplement: S2 Appendix — (DOCX) [file pone.0132922.s002.docx]

**Appendix 2 – Calculation of averted deaths**

Calculation of averted deaths

To calculate averted deaths we relied on a study that estimated influenza-associated excess mortality, and used vaccine effectiveness, vaccine coverage, and population statistics data, to calculate season and age-specific averted deaths [1].

Averted deaths were calculated according to the formula:

*Deaths averted = Number of excess deaths in the absence of vaccination x vaccine coverage x vaccine effectiveness*

Where

*Number of excess deaths in the absence of vaccination = number of excess deaths due to influenza under the current vaccination program/ proportion not protected by vaccination*

Which corresponds to

*Number of excess deaths in the absence of vaccination = number of excess deaths due to influenza under the current vaccination program/ (1 - vaccine coverage x vaccine effectiveness)*

Mortality information was retrieved from *National Center for Health Statistics*, and vaccination coverage data from the *National Health Immunization Survey.* Vaccine effectiveness data was taken from roughly the same studies used by Kostova et al [2].

Bibliography

[1]. Foppa IM, Cheng PY, Reynolds SB, Shay DK, Carias C, et al. (2015). Deaths averted by influenza vaccination in the U.S. during the seasons 2005/06 through 2013/14. Vaccine: forthcoming.

[2]. Kostova D, Reed C, Finelli L, Cheng PY, Gargiullo PM, et al. (2013) Influenza Illness and Hospitalizations Averted by Influenza Vaccination in the United States, 2005-2011. PLoS One 8: e66312.
