# Supplementary material for: Net Costs Due to Seasonal Influenza Vaccination — United States, 2005–2009
Source: PLoS One. 2015 Jul 31;10(7):e0132922. doi: 10.1371/journal.pone.0132922 (PMC4521706; doi:10.1371/journal.pone.0132922)
Supplement: S3 Appendix — (DOCX) [file pone.0132922.s003.docx]

**Appendix 3 – Additional inputs to calculate share of cases that was high-risk.**

Calculation of share of cases that was high-risk

The share of cases that was high-risk was obtained by dividing the share of high risk associated health outcomes, by the total number of health outcomes for death, outpatient visits, and hospitalizations [1] (Table SIII-1). For the 20-64 age-group, the mean was adapted from [1] taking into account the share of population in age categories 20 – 49 years old and 49 – 64 years old in 2009 (42 and 18%, respectively) [2]. In the sensitivity analyses this value was assumed to be 0 or 1, and the impact of these changes was analyzed.

Table SIII-1: Share of high risk associated health outcomes.

| Age-group | Share of high risk associated outcomes per age -group | | |
| --- | --- | --- | --- |
|  | Death | Hospitalization | Medically Attended Case |
| 0_4 | 0.05 | 0.05 | 0.09 |
| 5_19 | 0.10 | 0.11 | 0.20 |
| 20_64 | 0.21 | 0.21 | 0.33 |
| 65≥ | 0.51 | 0.51 | 0.58 |

Bibliography

[1]. Molinari NA, Ortega-Sanchez IR, Messonnier ML, Thompson WW, Wortley PM, et al. (2007) The annual impact of seasonal influenza in the US: measuring disease burden and costs. Vaccine 25: 5086-5096.

[2]. United States Census Bureau. American FactFinder: Age and Sex 2009 American Community Survey 1-Year Estimates. Available at: <http://factfinder.census.gov/faces/tableservices/jsf/pages/productview.xhtml?pid=ACS_11_1YR_S0101&prodType=table> . Accessed 4/1/2015.
