# Supplementary material for: Net Costs Due to Seasonal Influenza Vaccination — United States, 2005–2009
Source: PLoS One. 2015 Jul 31;10(7):e0132922. doi: 10.1371/journal.pone.0132922 (PMC4521706; doi:10.1371/journal.pone.0132922)
Supplement: S4 Appendix — (DOCX) [file pone.0132922.s004.docx]

**Appendix 4 – Additional inputs to estimate productivity losses, and cost of administering the vaccine**

Value of a lost day

The value of a lost day was calculated as the weighted average between the mean hourly wage times mean worked hours, and the value of an unspecified day, as calculated by [1]. That is:

*Value of a lost day of work = Mean hourly wage x mean worked hours x % employed population /100 + value of an unspecified day x % unemployed or out of the workforce population/100*

Where *% unemployed or out of the workforce population = 100 - % employed population*

Those <20 were assumed to require supervision by an individual older than 20 years old. For those 65≥, the share of unemployed or out of workforce population was adjusted. Inputs are shown in table SIV-1.

Table SIV-1: Inputs for calculation of the value of a lost day.

| Input | Value | Source |
| --- | --- | --- |
| Mean hourly wage (US$ 2009) | 20.90 | [2] |
| Mean worked hours per day (2013 data) | 8 | [3] |
| Value of an unspecified day (US$ 2009) | 107 | [1] |
| % Employed Population 20-64 years old (2014 data) | 73.5% | [4] |
| % Employed Population ≥65 years old (2014 data) | 17.7% | [4] |

Cost of administering the vaccine

The cost of administering the vaccine was equal to a weighted average between the cost of administering the vaccine in a medical setting (valued as a walk-in in a corporate clinic, $US 19.1) and non-medical setting (mass vaccination setting, estimated cost equal to $US 20.6) [5-8]. The weights correspond to the share of people being vaccinated in different settings, and vary per age group [7]. The share of people being vaccinated in a medical setting (non-medical setting) was: 54.4% (45.6%) for those 20 – 49 y., 56.3% (43.7%) for those 50 – 64 y., and 67.0% (33.0%) for those 65≥. The final value for the cohort 20-64 corresponded to a weighted average between the cohorts 20 – 49y and 50 – 64y, in which the weighs are the share of the population in each age bracket [4] (Table SIV.2).

The cost of administering the vaccine was assumed to follow a truncated normal distribution with standard deviation=mean/10, and values ranging from $US 14.0 (corresponding to the cost of administering the vaccine in a pharmacy) and $US 63.8 (corresponding to a scheduled doctor’s visit in a family size clinic) [5] [8].

Value of time lost to be vaccinated

To value the time lost to be vaccinated, we used data regarding time lost by influenza vaccine recipients in mass vaccination and medical settings retrieved from interviews and previously published [6]. Such cost was inflated to $US 2009 values using the Consumer Price Index (All Items component) [8]. The cost of time spent to be vaccinated in a medical setting was equal to:

*Cost of time spent to be vaccinated in a medical setting = Recipient time required x (Mean hourly wage x % Employed Population/100 + Value of an unspecified day/ Mean worked hours per day x (1-% Employed Population/100))*

Where Recipient time required was taken from [6], and was equal to 1.24 h for those 20 – 49y, 1.14 h for those 50 – 64 y, and 0.79 h for those 65≥. The final value for the cohort 20-64 corresponded to a weighted average between the cohorts 20 – 49y and 50 – 64y, in which the weighs are the share of the population in each age bracket [4].

Finally, the cost of time spent to be vaccinated was a weighted average between the cost of time spent to be vaccinated in a medical and non-medical setting (valued as time spent in a mass vaccination setting, estimated at $US 3.9 [6] [8]), where the weights corresponds to published survey data mentioned in the previous section [7].

The lower bound of time lost corresponds to the lower bound of the cost to be vaccinated in a mass vaccination setting ($US 2.2 [6] [8]), while the upper bound corresponds to assuming that vaccination occurs in a doctor’s office and requires 2 hours (valued as described in the previous formula) [7]. The value of the upper bound was calculated taking into account the different share of employed population for those <65 and 65≥ for the different age-groups [4].

The distribution assumed for medical and non-medical administration costs was normal with standard deviation=mean/10. Costs were truncated at the lower and upper bounds.

We further assumed all vaccinations took place during working hours.

Table SIV.2 – Vaccine administration cost ($US 2009)

| Item | Mean | Range |
| --- | --- | --- |
| Cost of administering the vaccine 0-64 | 19.8 | 14.0 – 63.8 |
| Cost of administering the vaccine 65≥ | 19.6 | 14.0 – 63.8 |
| Recipient time cost 0-64 | 14.7 | 2.2 – 37.6 |
| Recipient time cost 65≥ | 9.0 | 2.2 – 29.2 |

Bibliography

[1] Haddix AC, Teutsch SM, Corso PS (2003) Prevention effectiveness: a guide to decision analysis and economic evaluation: Oxford University Press.

[2] United States Department of Labor, Bureau of Labor Statistics. Occupational Labor Statistiscs - May 2009 National Occupational Employment and Wage Estimates United States. Available at: <http://www.bls.gov/oes/2009/may/oes_nat.htm#00-0000> . Accessed 4/1/2-15.

[3] United States Department of Labor, Bureau of Labor Statistics. American Time Use Survey —2013 Results (Table 4). Available at: <http://www.bls.gov/news.release/pdf/atus.pdf> . Accessed 4/1/2015.

[4] United States Department of Labor, Bureau of Labor Statistics. Labor Force Statistics from the Current Population Survey - 2014. Available at: <http://www.bls.gov/cps/cpsaat03.htm>. Accessed 4/1/2015.

[5] Coleman MS, Fontanesi J, Meltzer MI, Shefer A, Fishbein DB, Bennett NM, et al. Estimating medical practice expenses from administering adult influenza vaccinations. Vaccine. 2005;23: 915-923.

[6] Prosser LA, O'Brien MA, Molinari NA, Hohman KH, Nichol KL, Messonnier ML, et al. Non-traditional settings for influenza vaccination of adults: costs and cost effectiveness. Pharmacoeconomics. 2008;26: 163-178.

[7] Kennedy ED, Santibanez TA, Bryan LN, Wortley PM, Euler GL, Singleton JA, et al. Place of influenza vaccination among adults --- United States, 2010-11 influenza season. MMWR Morb Mortal Wkly Rep. 2011;60: 781-785.

[8] (2012) Statistical Abstract of the U.S. - Table 725. United States Census Bureau. Available: <http://www.census.gov/compendia/statab/2012edition.html>. Accessed: 4/1/2015.
